# IECP: iterative equilibration of cell-type expression profiles improves accuracy of reference-free deconvolution

**DOI:** 10.1093/bioinformatics/btag686

**Published:** 2026-09-15

**Authors:** Dongping Du, David M Herrington, Guoqiang Yu, Yue Wang, Yizhi Wang

**Affiliations:** Bradley Department of Electrical and Computer Engineering, Virginia Polytechnic Institute and State University, Arlington, VA 22203, United States; Department of Internal Medicine, Wake Forest University School of Medicine, Winston-Salem, NC 27157, United States; Department of Automation, Tsinghua University, Beijing 100084, P.R. China; Bradley Department of Electrical and Computer Engineering, Virginia Polytechnic Institute and State University, Arlington, VA 22203, United States; Bradley Department of Electrical and Computer Engineering, Virginia Polytechnic Institute and State University, Arlington, VA 22203, United States

## Abstract

**Motivation:**

Reference-free deconvolution methods are widely used to estimate cell-type composition and expression profiles from bulk transcriptomic data when native cell type references are unavailable. However, these methods often suffer from systematic biases caused by the asymmetric differential gene expressions across cell types and biological or experimental conditions, producing inaccurate proportion inference and reduced interpretability.

**Results:**

Here we present IECP (Iterative Equilibration of Cell-type Expression Profiles), an R package that improves deconvolution accuracy by iteratively equilibrating the asymmetric differential gene expressions across cell types. IECP identifies consistently expressed genes (CEGs) across estimated cell-type profiles, computes CEG-based sample-wise scaling factors, and equilibrates the bulk data matrix before the next deconvolution iteration. By integrating IECP with five popular reference-free deconvolution methods, CAM3.0, TOAST, PREDE, RefFreeEWAS, and CDseq, we demonstrate consistent improvements in cell-type proportion estimation on multiple benchmark datasets

**Availability:**

IECP R package is freely available at https://github.com/niccolodpdu/IECP, with sample data and application vignettes.

## 1 Introduction

Reference-free deconvolution methods have been developed to estimate cell type composition and expression from bulk data when reliable reference information is not available, yet are capable of identifying missing or new cell populations that would not have been considered a priori (Houseman *et al*. 2016, Li and Wu 2019, Qin *et al*. 2020, Karkar *et al*. 2024). Comprehensive evaluations of popular deconvolution methods have been reported using both in-silico mixtures of single-cell RNA-seq data and physical RNA mixtures of pure cell populations. These benchmarking studies have found that cell proportion estimates deviated from the true mixing proportions (Sutton *et al*. 2022, Dai *et al*. 2024). These studies also concluded that the observed deviations are due to biological and technical reasons, such as variability of cell-type signatures, missing reference cell types, and mismatch between single-cell derived references and native cell-type expressions in specific tissue microenvironment.

**Figure 1:**
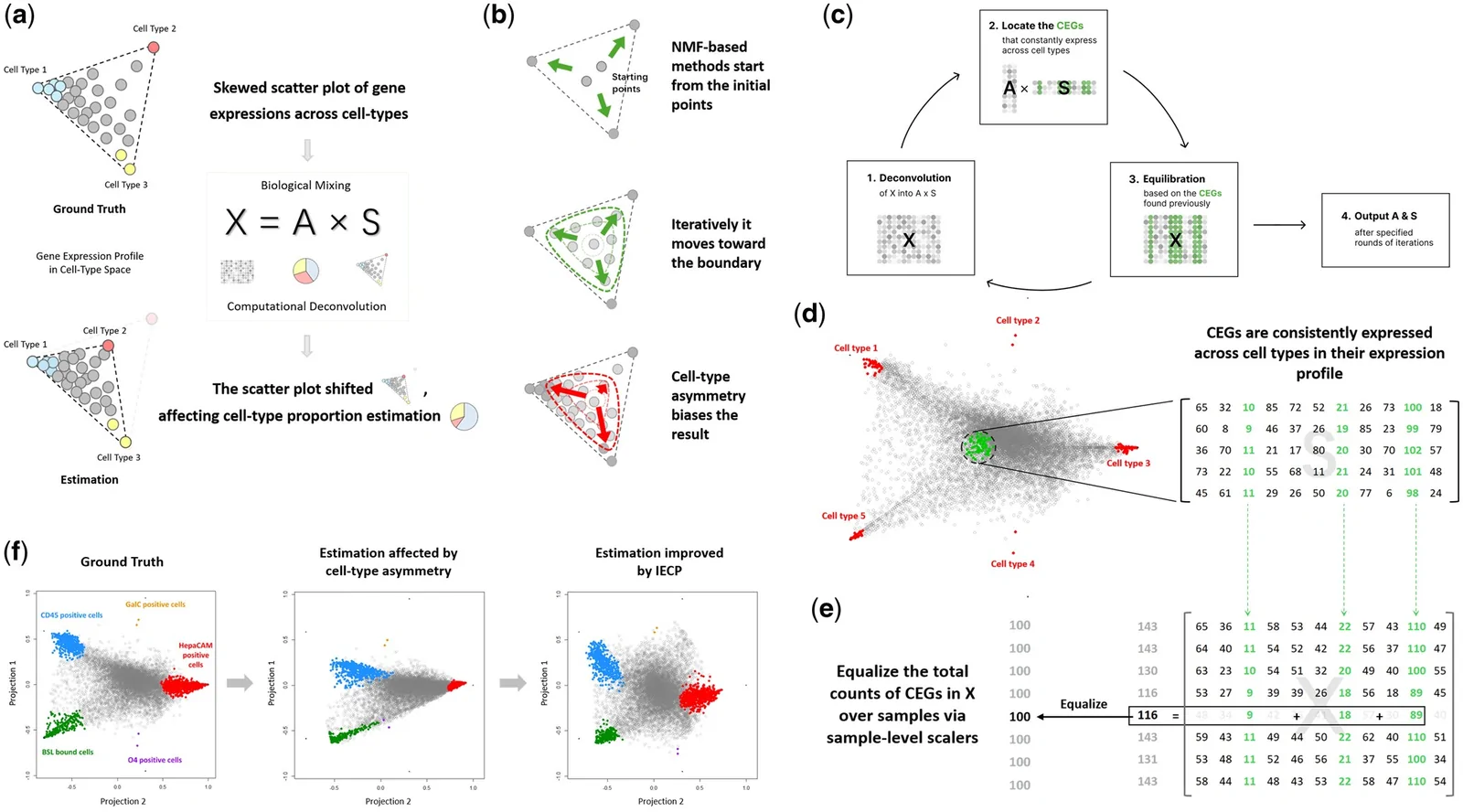
IECP pipeline and equilibration of cell-type asymmetry. (a) Illustration of asymmetric differential gene expressions using gene scatter plot in cell-type space. In ground truth, marker genes are correctly positioned while the scatter plot of gene expressions is uneven across cell types where most genes are clustering around the blue and yellow cell types. After biological mixing and deconvolution, the asymmetry isfurther propagated into estimation: marker of the red cell type becomes misaligned, affecting the proportionestimation. (b) Geometric explanation of how cell-type asymmetry affects NMF-rooted deconvolution, which can be interpreted as iteratively moving from initial points (top) toward the simplex boundary of the genescatter plot across cell types (middle). Cell-type asymmetry can severely misplace these boundary points and bias estimation (bottom). (c) IECP pipeline: (1) Deconvolute *X* into *A* and *S*; (2) Search for consistently expressed genes (CEGs) in *S*; (3) Equilibration based on CEGs; (4) Repeat steps 1–3 for certain iterations. (d) CEG identification via cosine similarity between each gene’s expression across cell types and an all-one vector. (e) Equilibration deriving sample-level scaling factors to equalize total CEG expression. (f) Example (GSE73721) shows that standard deconvolution skews gene distributions toward dominant cell types, while IECP improves the balance of marker-gene idealness across cell types and corrects the misplaced markers of particularly the cell types with less-ideal marker genes (O4 positive cells).

We found that this deviation of cell proportion estimation was at least partially the result of the asymmetric differential gene expressions among the constituent cell types (Evans *et al*. 2018, Wu *et al*. 2022), for example, skewed gene expression scatter plot in cell type space (see Supplementary Section 2.5, available as supplementary data at *Bioinformatics* online for detailed geometrical definition, projected visualization, and biological interpretation, and see Figs S1–S3, available as supplementary data at *Bioinformatics* online for examples) or unbalanced number of signature genes across cell types (Table S1–S3, available as supplementary data at *Bioinformatics* online). Deconvolution is widely modeled by a non-negative linear mixing process X=A×S, where X is the observed bulk expression matrix, A is the sample-wise cell-type proportion matrix, and S is the cell-type specific expression profile (Kuhn *et al*. 2011, Hart *et al*. 2015, Wang *et al*. 2016). Popular reference-free deconvolution methods are often rooted in the non-negative matrix factorization (NMF) framework (Qin *et al*. 2020, Karkar *et al*. 2024) while algorithmically implemented via different feature selection or optimization schemes (Sutton *et al*. 2022, Karkar *et al*. 2024). Here, we argue that the asymmetric differential gene expressions among the constituent cell types will affect reference-free deconvolution procedures and lead to inaccurate cell proportion estimation (Fig. 1a).

Based on the experimental results reported here (Table 1, Supplementary Data, available as supplementary data at *Bioinformatics* online), where popular reference-free deconvolution methods are thoroughly evaluated, we found that the asymmetric differential gene expression among the constituent cell types affects the performance of these reference-free deconvolution methods, broadly. To address it, we developed and evaluated a tailored plug-in software module, namely Iterative Equilibration of Cell-type expression Profiles (IECP), that iteratively improves deconvolution accuracy by leveraging consistently expressed genes (CEGs) as internal equilibration anchors. Conceptually, CEGs serve as reliable anchors and are used to equilibrate and reduce the potential bias introduced by asymmetric differential gene expressions across cell types. Practically, IECP progressively equalizes the contribution of each cell type in an iterative deconvolution process, thereby improving accuracy and robustness.

**Table 1:** Accuracy of the estimated proportion matrix, produced by five reference-free deconvolution methods with and without applying the IECP pipeline, on data simulated from GSE73721, initially normalized by total count normalization.

Methods	Cell-type correlation		Sample correlation		RMSE		Proportion angle deviation	
CAM3.0	0.905	0.921	0.877	0.899	0.174	0.139	13.86	13.44
RefFreeEwas	0.846	0.929	0.853	0.856	0.143	0.142	22.08	15.73
TOAST	0.844	0.897	0.885	0.905	0.175	0.131	22.64	19.85
PREDE	0.833	0.935	0.823	0.902	0.099	0.073	16.03	11.64
CDseq	0.831	0.885	0.596	0.789	0.115	0.089	14.18	12.96

Numbers underlined are associated with IECP.

## 2 Methods

### 2.1 Theoretical framework

For better interpretability, popular reference-free deconvolution methods principally adopt the NMF framework with solutions formulated as either constrained optimization or Bayesian estimation (Donoho and Stodden 2003, Gillis and Luce 2014, Kang *et al*. 2019). These methods are often equipped with integrated feature selection procedures (Houseman *et al*. 2016, Li and Wu 2019, Qin *et al*. 2020). To ensure identifiability, an accurate NMF-rooted solution requires features to be sufficiently dispersed across data scatter plot, and that there exist ideal cell-type marker genes (Laurberg *et al*. 2008, Ge and Zou 2015). In addition, other reference-free methods exploit the strong parallelism between a linear latent variable model and the theory of convex sets (Wang *et al*. 2016, Sutton *et al*. 2022, Karkar *et al*. 2024).

In practice the identifiability condition for reference-free deconvolution methods is often violated when the aforementioned asymmetric differential gene expressions are significant among constituent cell types, and thus results in an underinclusive deconvolution—particularly on cell types with few-ideal or less-ideal marker genes (Fig. 1b). IECP addresses this issue by iteratively equilibrating gene expression scatter plot across cell types, thereby restoring geometric symmetry, enhancing separability, and improving the estimation of cell-type proportions.

### 2.2 Overview of the IECP workflow

IECP provides an asymmetry equilibration module that integrates with existing reference-free deconvolution algorithms to address the biased proportion estimate. It functions as a wrapper around user-specified reference-free deconvolution methods. Each iteration of IECP consists of four main steps (Fig. 1c):

1. Deconvolution: The input bulk data matrix X is factorized into a cell-type proportion matrix A and a cell-type expression matrix *S* using any chosen reference-free deconvolution algorithm.
2. CEG identification: IECP identifies CEGs across the estimated cell-type profiles S (Fig. 1d).
3. Equilibration: IECP first calculates the CEG-based total count by summing the expression values of all CEGs in each sample, and creates the reference CEG total count by averaging individual CEG total counts over all samples. Then a scaling factor for each sample is determined by the ratio of reference CEG total count and the CEG total count in that sample, and applied to rescale every gene’s expression in that sample so that all samples share the same CEG total count (Fig. 1e).
4. Iteration: The equilibrated data are used as input for deconvolution in the next iteration, and the process is repeated until convergence or until a user-specified number of iterations is reached. Specifically, convergence is determined by the stability of the CEG index. When the subset of identified CEGs remains unchanged between consecutive iterations, the iterative equilibration process is considered to have reached a stationary point or converged. Two criteria can be used to accept the result by a cut-off based on: (i) the minimal reconstruction error measured by root mean square error (RMSE), or (ii) the maximal number of CEGs detected in *S*.

### 2.3 Identification of CEGs and equilibration

Accurate identification of CEGs is a key component in IECP that determines proper scaling factors to equilibrate asymmetric differential gene expressions across cell types. The cross–cell-type expression pattern of an ideal CEG (iCEG) is represented by the all-ones vector $\vec{1}$ (Evans *et al.* 2018). As in Cosbin (Wu *et al.* 2022), a cosine-based iCEG score quantifies the similarity between each gene’s expression profile x(i) and $\vec{1}$:


(#(1)) tiCEG(i)=cos⁡(x(i),1→)=∑j=1Kxj(i)K∑j=1K[xj(i)]2,

where K refers to the number of cell types and $1/\sqrt{K}<t_{\text{iCEG}}(i)<1$. Genes with high iCEG scores are selected as CEGs and used as reference features to equilibrate the bulk data X, ensuring equal total expression over these genes across samples.

CEGs serve as stable anchors for equilibrating the asymmetric differential gene expressions across cell types. Asymmetry across cell types is often associated with altered CEG gene expressions and total counts among cell types, and thus leads to altered CEG total counts in individual bulk samples under realistic cellular compositions. As a data-driven approach, IECP iteratively updates CEG content and total counts, calculates sample-wise scaling factors, and equilibrates bulk gene expressions, thus enforcing all samples share the same CEG total count.

### 2.4 Implementation

IECP is implemented as a modular R framework that can interface with any reference-free deconvolution algorithm returning the pair of proportion matrix A and cell-type expression profile S. Users may flexibly specify the underlying deconvolution algorithm, normalization procedure, and feature-selection method. The framework serves as a general wrapper that equilibrates outputs from existing reference-free methods while maintaining consistent normalization and feature-selection control.

#### 2.4.1 Practical plug-in usability

IECP is designed to function as an interoperable plug-in module that can be seamlessly incorporated into existing reference-free deconvolution workflows. It enhances estimation accuracy without modifying the original algorithms, normalization schemes, or preprocessing pipelines.

#### 2.4.2 Reproducible benchmarking

The accompanying R package provides complete benchmarking scripts, synthetic datasets, and demonstration vignettes to ensure transparency and full reproducibility of all computational experiments.

## 3 Results

We evaluated IECP using synthetic bulk data generated from three real-world datasets with varying degrees of cross cell-type asymmetric differential gene expressions: GSE73721 (human brain cell types), GSE220605 (breast cancer cell lines, monocytes, lymphocytes, and stem cells), GSE278443 (mouse mammary tumor cells and alveolar macrophages), and methylation data GSE110554. Cell-type proportion matrices were generated using Dirichlet distributions (*α* = 3), producing 30 samples per simulation. Both total count normalization and quantile normalization were applied to test robustness. We adopted synthetic data based experimental evaluation setting according to the common practice used by existing benchmark reports, because cell sorting may not provide an accurate ground truth yet few up-to-date in-vitro mix data are available in public domain (Sutton *et al*. 2022, Dai *et al*. 2024, Karkar *et al*. 2024). Note that realistic synthetic data provide both accurate ground truth and mixing diversity which are critical for the thorough evaluation of IECP-improved deconvolution performance.

To examine the applicability of IECP, we integrated it into five widely used reference-free deconvolution methods–CAM3.0 (Wang *et al*. 2016), TOAST (Li and Wu 2019), PREDE (Qin *et al*. 2020), RefFreeEWAS (Houseman *et al*. 2016), and CDseq (Kang *et al*. 2019)—and repeated each experiment 20 times with and without IECP. Four complementary metrics were used to quantify performance: (1) Cell-type correlation between estimated and ground-truth proportions (columns of proportion matrix), (2) Sample-wise correlation between estimated and true sample compositions (rows of proportion matrix), (3) RMSE of the estimated proportion matrix, and (4) Cell-type proportion angle deviation (columns of proportion matrix measure by angle degree).

Across all datasets, IECP consistently improved performance on all metrics (Table 1). For example, the cross cell-type asymmetric differential gene expressions are pronounced in GSE73721, IECP restored marker gene idealness (Kuhn *et al*. 2011, Wang *et al*. 2016), particular with the cell types with less-ideal marker genes that were lost in standard deconvolution (Fig. 1f). IECP also helped different methods in achieving a higher deconvolution accuracy across all metrics (Table 1). In GSE220605, IECP improved marker-gene idealness and eliminated false cell-type presence or inclusion. The improvements were consistent across different normalization and feature-selection procedures. Details of experimental results are available in Figs S7–S12 and Supplementary Data, available as supplementary data at *Bioinformatics* online. We also evaluated the performance of IECP on in-vitro mixture data (GSE19380 and GSE64385, Table S4, available as supplementary data at *Bioinformatics* online) and DNA methylation data (GSE110554, Fig. S13, available as supplementary data at *Bioinformatics* online). In both cases we observed consistent improvements. In addition, IECP is computationally efficient, requiring only modest additional runtime while delivering consistent accuracy improvements (Table S5, available as supplementary data at *Bioinformatics* online).

## 4 Discussion

Our experiments involving five reference-free deconvolution algorithms (CAM3.0, TOAST, PREDE, RefFreeEWAS, and CDseq), two initial normalization strategies (total-count and quantile normalization), three synthetic datasets with varying degrees of cross cell-type asymmetric differential gene expressions (GSE73721, GSE220605, GSE278443), two in-vitro mixture datasets (GSE19380 and GSE64385), and one DNA methylation dataset (GSE110554) demonstrate that IECP is broadly applicable across deconvolution frameworks, normalization methods and data types. It is compatible with different feature-selection procedures and effectively refines cell-type–specific marker genes under diverse asymmetry levels, leading to consistent improvements in proportion estimation accuracy. Across both total-count and quantile normalization settings, IECP consistently improves deconvolution accuracy, demonstrating its robustness to different initial normalization settings. While the evaluation here includes transcriptional and methylation data, the IECP framework and software tool are conceptually applicable to other omics data types as well.

There remain several limitations to IECP that should be considered. First, deconvolution accuracy improvement by IECP depends on the idealness of cell type marker genes (Donoho and Stodden 2003, Gillis and Luce 2014). Theoretically and experimentally, when ideal marker genes exist for every cell type, the expected improvement would be minimum. Second, IECP uses the all-ones vector $\vec{1}$ (Evans *et al*. 2018) as fixed reference to identify iCEGs. When cell type asymmetry is significant, a floating reference, e.g. the center of mass in the scatter plot may serve as a more sensible initial reference and thus should be exploited. Third, comprehensive evaluation is needed to assess the applicability and performance of IECP on different omics types, which could guide further development of IECP methodology.

## Supplementary Material

btag686_Supplementary_Data

## Data Availability

No new data were generated or analysed in support of this research. The public data used in this article are available in *Gene Expression Omnibus* with accession numbers GSE73721, GSE220605, GSE278443, and GSE110554.
