# Supplementary material for: IECP: iterative equilibration of cell-type expression profiles improves accuracy of reference-free deconvolution
Source: Bioinformatics. 2026 Sep 15;42(9):btag686. doi: 10.1093/bioinformatics/btag686 (PMC13616045; doi:10.1093/bioinformatics/btag686)
Supplement: Supplementary file 1 [file btag686_supplementary_data.zip › IECP_SI.docx]

Supplementary Information

**IECP: iterative equilibration of cell-type expression profiles improves accuracy of reference-free deconvolution**

Dongping Du, David M. Herrington, Guoqiang Yu, and Yue Wang, and Yizhi Wang

Contents

[1	Introduction	2](#_Toc236916422)

[1.1	Background	2](#_Toc236916423)

[1.2	Design Considerations	3](#_Toc236916424)

[2	Methods	3](#_Toc236916425)

[2.1	Rationale	3](#_Toc236916426)

[2.2	IECP Pipeline	4](#_Toc236916427)

[2.3	CEG Identification and Equilibration	5](#_Toc236916428)

[2.4	Performance Index	6](#_Toc236916429)

[2.5	Visualization of gene scatter plot in cell type space	7](#_Toc236916430)

[3	Simulation design	8](#_Toc236916431)

[4	Result	13](#_Toc236916432)

[4.1	Visualization of the effects of IECP	13](#_Toc236916433)

[4.2	Evaluation of IECP on synthetic bulk RNA-seq data	15](#_Toc236916434)

[4.3	Evaluation of IECP on in-vitro mixture data	22](#_Toc236916435)

[4.4	Evaluation of IECP on DNA Methylation Data	22](#_Toc236916436)

[4.5	Runtime and Memory Usage	24](#_Toc236916437)

[5	Discussion	25](#_Toc236916438)

[R Scripts & Demo	28](#_Toc236916439)

[Reference	28](#_Toc236916440)

# 1 Introduction

## 1.1 Background

The functions of complex tissues are orchestrated by a dynamic interaction among cell types, thus is of direct etiological interest to identify disease driving molecular and cellular archetypes and their compositional changes in native microenvironments (Lake, Ai et al. 2016, Herrington, Mao et al. 2018). Analyzing bulk gene expression data through cell-type level deconvolution can uncover patterns unique to the cell-cell and cell-matrix interactions within complex tissues (Wu, Du et al. 2024). Supervised methods estimate the component proportions using known gene-expression reference matrices, whereas reference-free deconvolution methods are often used to estimate cell type composition and specific gene expression from bulk samples when reliable reference information is not available (Kuhn, Thu et al. 2011, Newman, Liu et al. 2015, Hunt, Freytag et al. 2019). Supervised approaches are therefore limited by the quality of the reference signatures, while an intriguing advantage of unsupervised methods is that they can identify new cell populations or populations that would not have been taken into account a priori (Karkar, Sharma et al. 2024).

We and others have observed that differential gene expressions are often asymmetric, as closely examined via scatter plot of multiple cell types (Evans, Hardin et al. 2018). We were curious on how asymmetric differential gene expressions impact the accuracy of reference-free deconvolution of bulk data. While when ideal cell type marker genes (MGs) exist for every cell type(s) present in bulk data (e.g., Supplementary Fig S2), the methods rooted in convex analysis of mixtures principle theoretically immune from such a cell type asymmetry; when MGs are less-ideal or for methods relying on the interior gene expression profiles of cell types, the negative impact is suspected, particularly in the scenarios of imbalanced number of less-ideal MGs (e.g., Supplementary Fig S1 and S3). For example, NMF (non-negative matrix factorization) incorporated methods often include a feature selection step that can be heavily influenced by cell type asymmetry. More precisely, cell type asymmetry has an add-on impact on the selection of ‘informative genes’ being used in the deconvolution, leading to less-accurate estimate of cell type proportions. That is, the additional cause is the inaccurate selection of informative genes, that are altered by cell type asymmetry and subsequently impact deconvolution accuracy.

## 1.2 Design Considerations

Regarding whether ICA (independent component analysis) provides an alternative here, we would argue that ICA-based approaches are practically not suitable for reference-free deconvolution because the independence assumption is often violated in biological context (most cell types are highly likely correlated in gene expression space), yet ICA-derived components may also contain negative values that are difficult to interpret biologically. On the other hand, including Bayesian-based deconvolution method (e.g. CDseq) help demonstrate the broader applicability of IECP beyond standard NMF-incorporated approaches.

Initial normalization step is not necessarily required by the IECP iterative procedure, it was intentionally included to evaluate the robustness of IECP under different initial conditions. Specifically, we applied different normalization methods to input data prior to running IECP in order to test whether IECP could consistently improve deconvolution performance regardless of the initial data normalization strategy. Our results show that IECP remains effective across different initial normalization settings, indicating that the method is robust to variations in input scaling and normalization.

# 2 Methods

## 2.1 Rationale

Most reference-free deconvolution methods incorporate, either explicitly or implicitly, the NMF framework implemented via optimization or Bayesian approaches (Sutton, Poppe et al. 2022, Karkar, Sharma et al. 2024), where a unique NMF solution requires features to be sufficiently dispersed across the scatter simplex, often referred to as the separability or sufficiently scattered condition (Donoho and Stodden 2003, Laurberg, Christensen et al. 2008, Ge and Zou 2015). Mathematically, this separability is largely resolved by cell-type marker or signature genes, and the subsequent estimation accuracy is determined by the idealness of these marker or signature genes (Kuhn, Thu et al. 2011, Wang, Hoffman et al. 2016). However, differential gene expressions across cell types are often uneven as can be easily seen in the scatter plot (Supplement Fig S3). In addition, the number and idealness of cell type markers or signature genes are often imbalanced as well across cell types (Supplement Fig S1). Some cell types may contain many strong and specific marker genes, whereas others may contain fewer or less-specific markers. In this work, we refer to this imbalance as cell-type asymmetry. Such asymmetry can bias the contributions of individual cell types to the overall bulk data, where cell types with many ideal marker genes contribute much more variances than cell types with less-ideal marker or signature genes, thereby impairing accurate feature selection and proportion estimation in reference-free deconvolution.

We found it to be true through experiments that, despite what deconvolution model, feature selection method and normalization technique is applied, the cell-type asymmetry would always negatively affect the proportion estimation of cell types. Depending on the degree and form of asymmetry in the bulk data, the extent of deviation in proportion estimation varies across deconvolution models, feature selection strategies, and normalization techniques.

To address the negative impact of cell-type asymmetry on proportion inference, we developed the Iterative Equilibration of Cell-type expression Profiles (IECP) pipeline to assist NMF-based reference-free deconvolutions in the cell-type proportion inference under the influence of cell-type asymmetry.

## 2.2 IECP Pipeline

The IECP pipeline first deconvolutes the input bulk data matrix $X$ using a user-defined deconvolution method. Then IECP extracts the consistently expressed genes (CEGs) from the deconvoluted cell type specific expression profile $S$, conducts equilibration based on these CEGs. The input $X$ is then updated for the next round of iteration, as shown in **Figure 1**. The steps of IECP are as follows:

1. Deconvolution of $X$ into $A$ and $S$ by user-specific algorithm.
2. Search for CEG(s) in $S$.
3. Equilibration based on CEGs found in $S$.
4. Repeat step 1 - 3 until reaching the designated iteration count, and output $A$ and $S$.

In each round, IECP deconvolutes the mixed data matrix $X$ into proportion matrix $A$ and cell-type expression profile $S$ (step 1), searches for the CEGs across cell types in $S$ (step 2) and ensures the total count (numeric sum of expression) of CEGs are identical across samples in $X$ (step 3), gradually balancing the asymmetric cell types and recentering the overall gene distribution within the cell-type space.

In steps 2-3 we incorporate a CEG-based normalization strategy that was previously developed in Cosine score-based iterative normalization (Cosbin), which was originally used to balance and normalize biologically diverse samples. Compared to the classical Cosbin pipeline, we search for genes that are constantly expressed across cell types (step 2) instead of samples in $X$, and skip the step of eliminating asymmetric differentially expressed genes (DEGs) since the elimination of DEGs is only applicable when the total counts of samples are initially identical in the readout by principle, which is not necessarily true for the cell types in the deconvoluted $S$. Despite the fact that both Cosbin and IECP apply the scalers to the samples in the input data $X$, IECP tries to indirectly affect the gene locations in the cell-type space after deconvolution while Cosbin directly addresses the sample-wise differences in their expression level.

Two options for optimal solution are provided upon reaching the designated iteration steps (step 4): (1) minimum reconstruction error defined by Root Mean Square Error (RMSE), and (2) maximum number of CEGs found in $S$. Additionally, we allow users to integrate their preferred feature selection algorithms into the IECP pipeline (step 1), as some deconvolution algorithms require certain feature selection models as a part of their standard procedure.

## 2.3 CEG Identification and Equilibration

Mathematically, the cross-cell type expression pattern of an ideal CEG (iCEG) can be represented by the ‘all-ones’ vector $\vec{\boldsymbol{1}}$ (Evans, Hardin et al. 2018), which is used as the reference vector for identifying CEG(s). As in Cosbin (Wu, Shen et al. 2022), a cosine based iCEG score can be defined as

$$\begin{aligned} t_{\text{iCEG}}(i)=\cos\left( \boldsymbol{x}\left( i \right),\vec{\boldsymbol{1}} \right)=\frac{\sum_{j=1}^{K} x_{j}\left( i \right)}{\sqrt{K\sum_{j=1}^{K} \left[ x_{j}\left( i \right) \right]^{2}}},\boldsymbol{\#}\left( 1 \right) \end{aligned}$$

where $K$ refers to the number of cell types and $1/\sqrt{K}<t_{\text{iCEG}}(i)<1$. Based on this, IECP assigns each feature in the deconvoluted cell type specific expression profile $S$ with an iCEG score and chooses genes with iCEG scores higher than a given threshold as CEGs. IECP looks for CEGs that are constantly expressed across cell types, then uses these CEGs to equilibrate the bulk data $X$, ensuring that all samples have the same total count over these CEGs.

IECP derives sample-level scaling factors from the CEG expressions across samples (i.e., the total CEG counts per sample) and iteratively applies them to the bulk data to make sure the total CEG counts across all samples are equal. This iterative process of CEG identification and equalization progressively balances cell-type expression levels within the expression profile $S$. Because these scalers are applied to the entire bulk input matrix, the procedure also adjusts the expression levels of other genes, particularly differentially expressed genes or cell-type-specific marker genes (MGs) closely associated with the asymmetric cell type(s). In affected samples, these genes are systematically over- or underestimated, thus correcting these biases improves the accuracy of cell-type proportion estimation. In the most extreme cases, certain cell type(s) might become undetectable due to the unbalanced amount of differential expression, depending on the degree of such unbalance, the lack of cell-type-specific MG and the specific reference-free algorithm being used.

## 2.4 Performance Index

We used four quantitative measures to evaluate the improvement of proportion estimation by IECP: Pearson correlation coefficients on sample-level and cell-type-level, RMSE and angle distance. Specifically, RMSE is given by

$$RMSE= \sqrt{\frac{\sum_{\Omega} {(\hat{A}-A)}^{2}}{|A|}},$$

where $A$ is the ground truth proportion matrix, $|A|$ is the total number of entries, and $\hat{A}$ is the estimated proportion matrix (Oba, Sato et al. 2003, Stekhoven and Bühlmann 2012). Cell-type correlation coefficients and angle distances are calculated based on the cell-type specific proportions, namely the column vectors of $A$ and $\hat{A}$, and sample correlation coefficients are calculated by the row vectors of these two matrices.

## 2.5 Visualization of gene scatter plot in cell type space

Here we introduce gene expression scatter plot in cell-type space and how we visualize it in two-dimensional space (E.g., **Figure S1**). For brevity, we will omit the term “expression”.

In a gene scatter plot in cell-type space, each gene in the source matrix $S$ is represented by its expression vector across all $K$ cell types. To remove the effect of expression magnitude, the vector of each gene is divided by its total expression, so that each gene is mapped to a point in the $\left( K-1 \right)$-dimensional cell-type simplex. Ideal marker genes are located at the $K$ simplex vertices, whereas genes expressed in multiple cell types lie in the interior. For visualization, the high-dimensional simplex is mapped onto a two-dimensional space using the visualization procedures in CAM and debCAM (Chen, Wu et al. 2020, Wu, Du et al. 2024).

Specifically, the $K$ vertices of the simplex, each representing one cell type, are evenly placed at the boundary of a circle in a two-dimensional space. The corresponding coordinates will be (1,0), (cos(1/2π), sin(1/2π)), …, (cos(K-1)/2π, sin(K-1)/2π).The coordinate of each gene in this space is then calculated as the weighted average of the coordinates of $K$ vertices, where the weights are given by the normalized expression values of the gene across all cell types. Assume that a gene has expression $(s_{1},s_{2},\ldots,s_{K})$ across $K$ cell types, and $s_{1}+\ldots+s_{K}=1$, then its mapped coordinate will be:

$\left( s_{1}+cos\left( \frac{1}{2\pi} \right)s_{2}+\ldots+cos\left( \frac{K-1}{2\pi} \right)s_{K}, sin\left( \frac{1}{2\pi} \right)s_{2}+\ldots+sin\left( \frac{K-1}{2\pi} \right)s_{K} \right)$

Therefore, a gene mainly expressed in one cell type lies near the corresponding vertex, whereas a gene shared by multiple cell types lies in the interior of the simplex. The spatial distribution (dispersion) of genes reflects their cell-type specificity, and an asymmetric or skewed distribution indicates an imbalance in the number or specificity of marker genes across cell types.

# 3 Simulation design

The synthetic bulk data $X$ is generated by multiplying the cell type specific expression profiles extracted from real-world genomics data with a simulated sample-wise cell-type proportion matrix. The real-world genomics data we choose vary in the degree of cell-type asymmetry.

Our first set of cell type specific expression profiles are extracted from GSE73721, an expression profiling on RNA-Seq of human brain cells (Zhang, Sloan et al. 2016). Five cell types were extracted from the original data: HepaCAM positive cells (Astrocyte), GalC positive cells (Oligodendrocyte), CD45 positive cells (Myeloid), BSL bound cells (Endothelial), and O4 positive cells (Oligodendrocyte), all from adult temporal lobe. The original data contains several pure samples for each cell type, and the mean value of the relevant samples for expression profiles was used to form a cell-type expression matrix with the dimension of 15,041 (genes) × 5 (cell types). For illustration, we used COT (Lu, Wu et al. 2022) to identify the marker genes for each cell type. COT is a tool we previously developed based on cosine value thresholding of features. To showcase the asymmetry among cell types, we selected the top 1,500 features from COT result. **Table S1** suggests that three of the cell types (HepaCAM positive cells, CD45 positive cells and BSL bound cells) have significantly higher numbers of marker genes than the other two (GalC positive cells and O4 positive cells). Moreover, as suggested in **Figure S1** by the distance between a certain marker gene from the vertices of that cell type (triangle in the plot, ideal marker gene that uniquely expressed in that cell type), GalC positive cells and O4 positive cells have significantly weaker marker genes than the rest of the cell types.

**Figure S1.** Gene scatter plot in the cell-type space for cell type specific expression profile extracted from GSE73721. Each dot is the expression of one gene across all cell types projected to 2D. Marker genes for each cell type are identified by COT and are colored accordingly. Triangles at the vertices are the ideal marker genes that uniquely express in a certain cell type, used as the coordinates for the cell-type space.

**Table S1.** Number of marker genes for each cell type in cell type specific expression profile extracted from GSE73721

**Figure S2.** Gene scatter plot in the cell-type space for the cell type specific expression profile extracted from GSE220605.

**Table S2.** The number of marker genes for each cell type in the cell type specific expression profile extracted from GSE220605.

**Figure S3.** Gene scatter plot in the cell-type space for the cell type specific expression profile extracted from GSE278443.

**Table S3.** Number of marker genes for each cell type in the cell type specific expression profile extracted from GSE278443

The second set of cell-type specific expression profiles are extracted from GSE220605. In this dataset cells from 3 breast cancer lines (T47D, BT474, MCF7), monocytes (Thp1), lymphocytes (Jurkat), and stem cells (hMSC) were profiled by bulk RNA-seq in triplicates (Avila Cobos, Vandesompele et al. 2018). Mean values were calculated for each record without missing value. The final output is a cell-type expression matrix with the dimension of 36,552 (genes) × 6 (cell types). Notably, in a total of 1,000 marker genes, lymphocytes (Jurkat) have the highest number of marker genes, almost twice the number for T47D, which indicates the asymmetry among cell types. We again used scatter plot of cell-type space and COT to examine the possible asymmetry of other cell types for the expression profiles:

The third cell type specific expression profiles are from GSE278443, a bulk RNA-sequencing data on mouse mammary tumor cells and alveolar macrophages. The three cell types we extracted include mammary early lesion cells, mammary primary tumor cells and lung alveolar macrophages, all from mono-culture controls (Dalla, Papanicolaou et al. 2024). Mean values were calculated for each triplicated record without any missing value. The final cell type specific expression matrix has 12,726 genes in 3 cell types. Similarly, we plotted the distribution of the gene expression profiles in the cell-type space (**Figure S3**.) and used COT for marker gene selection (**Table S3.**). Notably, the mammary primary tumor cell type has both significantly less marker genes and lower density of genes around its corner.

The sample-wise cell-type proportion matrix is simulated by the Dirichlet distribution function in R. Dirichlet distribution allows an overall balanced cell-type proportion among samples without assuming the existence of ideally pure samples of certain cell types, as in most cases pure samples are not available or unknown for the reference-free deconvolution. We set α (the parameter controlling the shape of the distribution) at 3 for all cell types in our simulation to make sure the proportions among samples are basically concentrated towards the center and mutually symmetric. 30 samples are generated for each experiment.

To test the robustness of IECP under different initial normalization conditions, we separately applied two commonly used approaches, total count normalization and quantile normalization, to the input bulk data prior as part of the pre-processing.

To validate the applicability of IECP on different reference-free deconvolution algorithms, we included five popular models: CAM3.0 , TOAST (Li and Wu 2019), PREDE (Qin, Zhang et al. 2020), RefFreeEWAS (Houseman, Kile et al. 2016), and CDseq (Kang, Meng et al. 2019). These models vary in their algorithm design and all incorporate their own specific feature selection units as a part of their procedure, which were integrated into the IECP pipeline. Bulk data input matrix was generated by mixing the cell-type specific expression profiles $S$ extracted from real-world data (GSE73721, GSE220605, GSE278443, GSE110554) with a Dirichlet distributed cell-type proportion matrix $A$ following $X=A\times S$. Total count normalization and quantile normalization were separately applied prior to experiments. For each deconvolution algorithm, experiments were performed 20 times, with and without using the IECP pipeline. Four performance metrics were applied: cell-type correlation coefficients, sample-wise correlation coefficients, RMSE and cell-type angle distance, to quantify the improvement of proportion estimation by applying IECP to these deconvolution methods.

# 4 Result

## 4.1 Visualization of the effects of IECP

The following gene scatter plots are depicted based on the cell-type specific expression profiles in the cell-type space that are separately deconvoluted by CAM3.0, PREDE, TOAST and RefFreeEwas from the input bulk matrix, which follows the procedure mentioned in the previous section and normalized by total count normalization, with and without applying the IECP pipeline. Marker genes for each cell type are identified by COT from the ground truth cell type specific expression profiles and are colored in each plot accordingly.

In **Figure S4.** RefFreeEWAS produced a markedly distorted cell-type expression profile, likely because variance-based feature selection failed to capture HepaCAM-positive cells (red), whereas IECP successfully retrieved these markers. TOAST resulted in a compressed profile skewed toward major cell types, but IECP yielded a more balanced distribution closely matching the ground truth. PREDE also biased toward major cell types, misplacing minor ones within dominant marker-gene space, leading to their loss; IECP recovered these markers, particularly for GalC-positive cells (yellow).

Similar patterns can be found with the simulation data generated from GSE220605. **Figure S5.** shows that IECP helped RefFreeEWAS remove one false positive cell type on the top right corner. With IECP, TOAST found back the missing cell type HMSC colored in pink, and for PREDE the marker-gene quality for MCF7 (colored in blue) significantly improved.

**Figure S4.** Gene scatter plot in the cell-type space, with the simulation data generated from GSE73721.

**Figure S5.** Gene scatter plot in the cell-type space, with the simulation data generated from GSE220605.

**Figure S6.** Gene scatter plot in the cell-type space, with the simulation data generated from GSE278443.

**Figure S6.** suggests that, for RefFreeEWAS, TOAST and PREDE, IECP helped in ruling out the false positive cell types (RefFreeEWAS: bottom-right corner; TOAST: top-right corner; PREDE: middle-left).

## 4.2 Evaluation of IECP on synthetic bulk RNA-seq data

The following boxplots illustrate the improvement achieved by IECP in proportion estimation across four metrics: cell-type correlation coefficients, sample-wise correlation coefficients, RMSE, and cell-type angle distance. Input bulk matrices are normalized by total count (**Figure S7 to Figure S9**) or quantile normalization (**Figure S10 to Figure S12**). The result shows that IECP steadily improves both sample-wise and cell-type-wise correlation coefficients and reduces RMSE and cell-type angle distances under all scenarios. In addition to optimization-based NMF approaches, we also included CDSeq, a popular and representative Bayesian-based deconvolution method, in our evaluation.

**Figure S7.** Accuracy of the estimated proportion matrix from GSE73721 by five reference-free deconvolution methods (CAM3.0, RefFreeEwas, PREDE, TOAST, and CDSeq) with and without applying the IECP pipeline. The input bulk data was normalized by total count normalization. Top left: cell type correlation coefficient (higher is better). Top right: sample-wise correlation (higher is better). Bottom left: RMSE (lower is better). Bottom right: angle between ground truth and estimated proportion (lower is better). The mean, median, and maximum value of each box plot is given below each subplot.

**Figure S8.** Accuracy of the estimated proportion matrix from GSE220605 by five reference-free deconvolution methods with and without applying the IECP pipeline. The input bulk data was normalized by total count normalization.

**Figure S9.** Accuracy of the estimated proportion matrix from GSE278443 by five reference-free deconvolution methods with and without applying the IECP pipeline. The input bulk data was normalized by total count normalization.

**Figure S10.** Accuracy of the estimated proportion matrix from GSE73721 by five reference-free deconvolution methods with and without applying the IECP pipeline. The input bulk data was normalized by quantile normalization.

**Figure S11.** Accuracy of the estimated proportion matrix from GSE220605 by five reference-free deconvolution methods with and without applying the IECP pipeline. The input bulk data was normalized by quantile normalization.

**Figure S12.** Accuracy of the estimated proportion matrix from GSE278443 by five reference-free deconvolution methods with and without applying the IECP pipeline. The input bulk data was normalized by quantile normalization.

To evaluate the statistical significance of the performance improvements introduced by IECP, we performed Wilcoxon tests comparing deconvolution results obtained with and without IECP. The corresponding results are reported in the Supplementary Data.

## 4.3 Evaluation of IECP on in-vitro mixture data

Experimentally generated in vitro mixture datasets offer complementary validation under more realistic biological settings. Therefore, to further evaluate IECP’s performance, we included two in vitro mixture datasets, GSE19380 and GSE64385, both generated from experimentally mixed purified cell populations with known mixing proportions. CAM3.0 was used in this experiment. We opt not to include other deconvolution methods here because CAM3.0 provides good baseline performance for evaluation, and IECP shows similar impact across different reference-free deconvolution methods on the synthetic data. As shown on **Table S4**, IECP consistently improved the estimation accuracy on both datasets. However, it should be noted that the reported mixing proportions or cell-sorting estimates in in-vitro experiments may still deviate from the true cellular compositions due to experimental and technical variability or imperfection. Therefore, we regard these datasets as providing complementary evidence about the utility of IECP rather than definitive benchmarks. Nevertheless, the comprehensive evaluation on in-vitro mixture and cell sorting datasets will be part of our future research.

| Method | Dataset | Metric | Without IECP | With IECP | Improvement (%) |
| --- | --- | --- | --- | --- | --- |
| CAM3.0 | GSE19380 | Cell-type correlation | 0.8683 | 0.9851 | 13.45 |
|  |  | Sample correlation | 0.9617 | 0.966 | 0.45 |
|  |  | RMSE | 0.4584 | 0.1573 | 65.68 |
|  |  | Proportion Angle Deviation | 29.55 | 17.1 | 42.13 |
|  | GSE64385 | Cell-type correlation | 0.9721 | 0.9793 | 0.74 |
|  |  | Sample correlation | 0.85 | 0.8573 | 0.86 |
|  |  | RMSE | 0.0813 | 0.077 | 5.29 |
|  |  | Proportion Angle Deviation | 11.24 | 10.42 | 7.3 |

Table S4. Evaluation of IECP on GSE19380 and GSE64385

## 4.4 Evaluation of IECP on DNA Methylation Data

To further evaluate the applicability of IECP on different omics data types, we evaluated ICEP on the DNA methylation dataset GSE110554, which contains purified human blood cell populations profiled using the Illumina HumanMethylation450 platform. Cell-type-specific methylation profiles were extracted to generate synthetic bulk methylation samples following the same simulation procedure described above.

**Figure S13.** Accuracy of the estimated proportion matrix from DNA methylation data GSE110554 by three reference-free deconvolution methods with and without applying the IECP pipeline.

We included TOAST, RefFreeEWAS and CAM3.0 in this evaluation, and compared deconvolution accuracy with and without the assistance of IECP for all three methods. The results are summarized in **Figure S13** and the Supplementary Data. As shown in **Figure S13**, IECP consistently improves the deconvolution accuracy for all these three methods on this DNA methylation data.

## 4.5 Runtime and Memory Usage

To estimate the computational overhead introduced by IECP, we recorded runtime and peak memory usage during the deconvolution experiments. RefFreeEWAS was used as the embedded deconvolution algorithm, and measurements were obtained with and without IECP under identical simulation settings. The results are reported in **Table S4.** and in the Supplementary Data. They indicate that IECP improves deconvolution performance while introducing only modest additional computational cost.

**Table S5.** Runtime and peak memory usage of IECP. Runtime and peak memory usage were measured using RefFreeEWAS with and without IECP under identical simulation settings. “Runtime: RefFreeEWAS × Iteration #” represents the estimated runtime of repeated RefFreeEWAS deconvolution without IECP, whereas “Runtime: IECP” denotes the total runtime of the IECP pipeline. The additional runtime introduced by IECP per iteration was calculated as $(\text{Runtime: IECP}-\text{Runtime: RefFreeEWAS}\times\text{Iteration \#})/\text{Iteration \#}$. Memory usage corresponds to the peak memory consumption recorded during execution.

# 5 Discussion

From our experiments that involved five reference-free deconvolution algorithms (CAM3.0, TOAST, PREDE, RefFreeEWAS, CDseq), different embedded feature selection techniques, two normalization conditions (total count normalization, quantile normalization) and four sets of data that range in the degrees of cell-type asymmetry (GSE73721, GSE220605, GSE278443) or data source (GSE110554, DNA methylation data), IECP is proven to be applicable to a range of deconvolution methods for proportion estimation, compatible with different normalization and feature selection techniques, and able to target and refine the cell-type specific markers under different degrees of cell-type asymmetry embedded in the bulk data, leading to a visible improvement of accuracies in proportion estimation in these scenarios.

The influence of cell-type asymmetry can be interpreted through the geometric perspective commonly used in NMF and related reference-free deconvolution methods, such as convex analysis of mixture. In these approaches, genes are represented in a cell-type expression space, where cell-type-specific marker genes tend to define the boundaries and vertices associated with distinct cell-type signals. Accurate deconvolution therefore depends on sufficiently informative marker-gene representation across all cell types (Donoho and Stodden 2003, Gillis and Luce 2014, Ang and Gillis 2019). However, when marker genes are unevenly distributed across cell types, certain cell types become strongly represented while others are supported by fewer, weaker, or less specific markers. This imbalance distorts the expression structure and makes weakly represented cell types more difficult to distinguish during deconvolution. As shown in **Figure S4–S6**, cell-type asymmetry substantially affects the positioning and recovery of marker genes, whereas IECP mitigates these distortions through iterative equilibration.

This interpretation is consistent with theoretical studies on identifiability in NMF and related simplex-based matrix decomposition methods. Laurberg et al. showed that accurate and unique NMF solutions require features to be sufficiently dispersed so that each latent component can be reliably distinguished (Laurberg, Christensen et al. 2008). A stronger version of this requirement is the separability assumption, in which the vertices associated with latent components are explicitly represented in the observed data matrix (Donoho and Stodden 2003). While some algorithms, such as fast recursive NMF, directly rely on separability (Gillis and Vavasis 2013), many related approaches including subset-NMF, Maximum Volume Inscribed Ellipsoid (MVIE), and Minimum Volume Enclosing Simplex (MVES) instead adopt weaker geometric assumptions, such as sufficiently scattered or properly distributed feature structures (Ge and Zou 2015, Lin, Ma et al. 2015, Huang, Fu et al. 2016, Lin, Wu et al. 2018).

Although these assumptions differ mathematically, they share a common requirement that each latent component must be supported by sufficiently informative and distinguishable features. In the context of cell-type deconvolution, this means that each cell type should contribute enough strong and specific marker genes to define distinguishable regions in the cell-type expression space. Cell-type asymmetry violates this requirement by causing certain cell types to be represented by fewer, weaker, or less specific marker genes, thereby making them more difficult to distinguish and accurately estimate during deconvolution. By iteratively equilibrating cell-type expression profiles, IECP improves the balance of marker-gene representation across cell types and consequently improves the stability and accuracy of reference-free deconvolution.

While IECP aims to equilibrate asymmetric cell-type expression profiles, it relies on CEGs being consistently expressed across all cell types. This requirement helps preserve biological interpretability during equilibration and reduces the risk of over-correcting samples associated with weakly represented cell types, which could otherwise introduce artificial cell-type structures during deconvolution. However, severe cell-type asymmetry may itself interfere with accurate CEG identification. IECP therefore iteratively updates CEG selection and sample equilibration rather than relying on a single round of correction.

The levels and forms of cell-type asymmetry will affect the performance of IECP. In our experiments, we separately extracted three sets of cell-type specific expression profiles from GSE73721, GSE220605 and GSE278443 that vary in the levels of cell-type asymmetry in terms of amount of differential expression and marker-gene idealness across cell types (**Figure S1. -** **S3.**, **Table S1. - S3.**). In the experiments, depending on the levels of asymmetry, IECP assisted the corresponding reference-free deconvolution algorithm in recovering the lost marker genes (**Figure S4. & S5.**), refining the location of marker genes in the cell-type space (**Figure S4. &** **S5.**), ruling out false positive ones (**Figure S6.**) or improving their qualities (**Figure S4.**). However, it is still possible that when extreme asymmetry of cell-types exists, IECP could not retrieve certain lost cell type(s) (**Figure S4.**).

An important practical consideration of IECP is the computational trade-off introduced by iterative equilibration. Because IECP repeatedly performs deconvolution, CEG identification, and sample-wise rescaling, the computational cost increases with the number of iterations and depends on the complexity of the underlying deconvolution algorithm (**Table S4.**). In addition, the extent of improvement achieved by IECP depends on the severity of cell-type asymmetry: moderate asymmetry can often be substantially corrected, whereas extremely weakly represented cell types, characterized by very few or weak marker genes, may remain difficult to recover.

Supervised deconvolution currently falls outside the scope of IECP. When accurate reference profiles are available, cell-type-specific marker information is already externally constrained, reducing the dependence of deconvolution on the structure of the bulk data itself. Depending on the type of supervision and the algorithm used, the challenges of asymmetry in marker gene detection may be alleviated or even bypassed. If accurate cell-type expression profiles are provided, iterative CEG identification and equilibration may no longer be required for proportion estimation.

In our experimental evaluation, we have found that in some cases, IECP did not noticeably improve the deconvolution accuracy by CAM3.0, where the expression patterns of detected marker genes are used to estimate cell type proportions. By a closer look at the cell types that are involved, the marker genes are either ideal or close-to-ideal. In relation to the estimation of cell type proportions, it can be shown that the expression patterns of ideal MGs (directions of column vectors in proportion matrix) are not theoretically impacted by asymmetric differential gene expressions. Practically, when marker genes are less-ideal yet cell type asymmetry is significant, IECP will likely improve the deconvolution accuracy by CAM3.0.

In the experiment, we applied IECP on the deconvolution of DNA methylation data. However, we should emphasize that that experiment mainly shows the applicability of IECP on methylation data and is not a comprehensive evaluation. Different omics data types may have different characteristics, and the application of IECP beyond expression data needs careful evaluation and potential modification of the algorithm. For example, unlike expression data, the distribution of methylation data is very different, which shows a characteristic bi-modal pattern, which may alter the pattern in the gene scatter plot and thus influence the performance of IECP. One future direction of IECP development is to perform a comprehensive evaluation on different omics types and potentially consider the special properties of each data type to improve the performance of IECP.

# R Scripts & Demo

The IECP pipeline is open-source and available at [https://github.com/niccolodpdu/IECP](https://github.com/niccolodpdu/ITEA).

# Reference

Ang, A. M. S. and N. Gillis (2019). "Algorithms and comparisons of nonnegative matrix factorizations with volume regularization for hyperspectral unmixing." IEEE Journal of Selected Topics in Applied Earth Observations and Remote Sensing **12**(12): 4843–4853.

Avila Cobos, F., et al. (2018). "Computational deconvolution of transcriptomics data from mixed cell populations." Bioinformatics **34**(11): 1969–1979.

Chen, L., et al. (2020). "debCAM: a bioconductor R package for fully unsupervised deconvolution of complex tissues." Bioinformatics **36**(12): 3927–3929.

Dalla, E., et al. (2024). "Lung-resident alveolar macrophages regulate the timing of breast cancer metastasis." Cell **187**(23): 6631–6648.e6620.

Donoho, D. and V. Stodden (2003). "When does non-negative matrix factorization give a correct decomposition into parts?" Advances in Neural Information Processing Systems **16**.

Evans, C., et al. (2018). "Selecting between-sample RNA-Seq normalization methods from the perspective of their assumptions." Brief Bioinform **19**(5): 776–792.

Ge, R. and J. Zou (2015). Intersecting faces: Non-negative matrix factorization with new guarantees. International Conference on Machine Learning, PMLR.

Gillis, N. and R. Luce (2014). "Robust near-separable nonnegative matrix factorization using linear optimization." The Journal of Machine Learning Research **15**(1): 1249–1280.

Gillis, N. and S. A. Vavasis (2013). "Fast and robust recursive algorithmsfor separable nonnegative matrix factorization." IEEE transactions on pattern analysis and machine intelligence **36**(4): 698–714.

Herrington, D. M., et al. (2018). "Proteomic Architecture of Human Coronary and Aortic Atherosclerosis." Circulation **137**(25): 2741–2756.

Houseman, E. A., et al. (2016). "Reference-free deconvolution of DNA methylation data and mediation by cell composition effects." BMC Bioinformatics **17**: 259.

Huang, K., et al. (2016). "Anchor-free correlated topic modeling: Identifiability and algorithm." Advances in Neural Information Processing Systems **29**.

Hunt, G. J., et al. (2019). "Dtangle: accurate and robust cell type deconvolution." Bioinformatics **35**(12): 2093–2099.

Kang, K., et al. (2019). "CDSeq: A novel complete deconvolution method for dissecting heterogeneous samples using gene expression data." PLoS Comput Biol **15**(12): e1007510.

Karkar, S., et al. (2024). "DECOMICS, a shiny application for unsupervised cell type deconvolution and biological interpretation of bulk omic data." Bioinform Adv **4**(1): vbae136.

Kuhn, A., et al. (2011). "Population-specific expression analysis (PSEA) reveals molecular changes in diseased brain." Nat Methods **8**(11): 945–947.

Kuhn, A., et al. (2011). "Population-specific expression analysis (PSEA) reveals molecular changes in diseased brain." Nat Methods **8**(11): 945–947.

Lake, B. B., et al. (2016). "Neuronal subtypes and diversity revealed by single-nucleus RNA sequencing of the human brain." Science **352**(6293): 1586–1590.

Laurberg, H., et al. (2008). "Theorems on positive data: On the uniqueness of NMF." Computational intelligence and neuroscience **2008**(1): 764206.

Li, Z. and H. Wu (2019). "TOAST: improving reference-free cell composition estimation by cross-cell type differential analysis." Genome Biol **20**(1): 190.

Lin, C.-H., et al. (2015). "Identifiability of the simplex volume minimization criterion for blind hyperspectral unmixing: The no-pure-pixel case." IEEE Transactions on Geoscience and Remote Sensing **53**(10): 5530–5546.

Lin, C.-H., et al. (2018). "Maximum volume inscribed ellipsoid: A new simplex-structured matrix factorization framework via facet enumeration and convex optimization." SIAM Journal on Imaging Sciences **11**(2): 1651–1679.

Lu, Y., et al. (2022). "COT: an efficient and accurate method for detecting marker genes among many subtypes." Bioinformatics Advances **2**(1): vbac037.

Newman, A. M., et al. (2015). "Robust enumeration of cell subsets from tissue expression profiles." Nature methods **12**(5): 453–457.

Oba, S., et al. (2003). "A Bayesian missing value estimation method for gene expression profile data." Bioinformatics **19**(16): 2088–2096.

Qin, Y., et al. (2020). "Deconvolution of heterogeneous tumor samples using partial reference signals." PLOS Computational Biology **16**(11): e1008452.

Stekhoven, D. J. and P. Bühlmann (2012). "MissForest—non-parametric missing value imputation for mixed-type data." Bioinformatics **28**(1): 112–118.

Sutton, G. J., et al. (2022). "Comprehensive evaluation of deconvolution methods for human brain gene expression." Nat Commun **13**(1): 1358.

Wang, N., et al. (2016). "Mathematical modelling of transcriptional heterogeneity identifies novel markers and subpopulations in complex tissues." Scientific reports **6**: 18909.

Wu, C.-T., et al. (2024). "CAM3. 0: determining cell type composition and expression from bulk tissues with fully unsupervised deconvolution." Bioinformatics **40**(3): btae107.

Wu, C.-T., et al. (2022). "Cosbin: cosine score-based iterative normalization of biologically diverse samples." Bioinformatics Advances **2**(1): vbac076.

Zhang, Y., et al. (2016). "Purification and characterization of progenitor and mature human astrocytes reveals transcriptional and functional differences with mouse." Neuron **89**(1): 37–53.
